# Platelet-rich fibrin and collagen matrix for the regeneration of infected necrotic immature teeth

**Published:** 2020-07-08

**Authors:** S. Sakthivel, V. Gayathri, Subha Anirudhan, R. Jaya Shree Roja

**Affiliations:** ^1^Department of Conservative Dentistry and Endodontics, Sri Ramakrishna Dental College and Hospital, Coimbatore, Tamil Nadu, India; ^2^Department of Conservative Dentistry and Endodontics, Sri Ramakrishna Dental College and Hospital, SNR Road, Coimbatore, Tamil Nadu, India

**Keywords:** collagen matrix, mineral trioxide aggregate, platelet-rich fibrin, regeneration

## Abstract

Revitalization of an immature necrotic tooth is possible under conditions of total canal disinfection. This case report describes an immature tooth #21 with periapical pathology which was treated through revascularization using 3% sodium hypochlorite and 17% ethylenediaminetetraacetic acid as irrigants; calcium hydroxide was placed as an intracanal medicament, platelet-rich fibrin as a scaffold, and collagen matrix was placed as a barrier. On follow-up at 2 years, healing of periapical lesion, apical closure could be appreciated. Based on the results, we conclude that regeneration of necrotic tooth is possible under efficient disinfection.

**Relevance for Patients:**

This article documents the 2-year outcome of regenerative endodontic treatment in necrotic immature teeth. The successful outcome of this case shows that regenerative endodontic teeth can be a viable treatment option in immature teeth.

## 1. Introduction

The most challenging canals to obturate three-dimensionally are those with open apices. Closure of their apices, using apexification techniques have been traditionally tried with calcium hydroxide, and later, mineral trioxide aggregate (MTA). However, with these methods, the remaining dentinal walls remain thin and are predisposed the tooth to fracture during function [1]. A much desirable result would be to achieve regeneration of the healthy pulp dentin complex resulting in the strengthening of the roots.

Regeneration, also referred to as revascularization, results in apical closure and thickening of the lateral dentinal walls along with regeneration of pulp dentin complex [2]. It paves the way for maturogenesis in the non-vital immature tooth. The remnant viable cells in the apical cells can proliferate and aid in the process of regeneration [3]. This facilitation of root development might increase the structural strength of the tooth and hence improve the chances of its survival. Stem cells, scaffolds, and growth factors are three important requirements for the success of this procedure [4].

The stem cells involved in the process are mainly stem cells of the apical papilla, periodontal ligament, pulp, and bone marrow. A depleted canal will not stake cell growth. Hence, a scaffold is essential to support cell organization and vascularization [5].

Literature reveals that there are very few cases documenting the application of platelet-rich fibrin (PRF) in the field of regenerative endodontics. Here, we present a case of an immature upper lateral incisor with periapical pathology wherein regenerative endodontic procedure was undertaken.

## 2. Case Presentation

A 14-year-old male patient reported with his parents to the Department of Conservative Dentistry and Endodontics, with the chief complaint of broken restoration and loosening of teeth in the upper front tooth region on July 10, 2017. The patient gave a history of trauma of the front teeth 5 years back for which a tooth-colored restoration had been placed. His medical history was not contributory. There were no significant signs of pathosis on the extraoral examination. On intraoral examination, it was evident that tooth #21 had an uncomplicated crown fracture with grade I mobility. There were no soft-tissue abnormalities.

**Figure 1:**
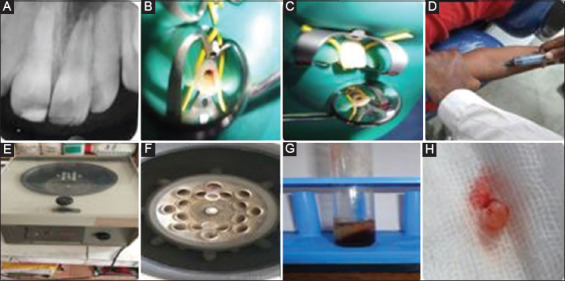
A. Pre-operative radiograph, B. Access opening, C. After calcium hydroxide removal, D. Blood drawn from anterior cubital vein, E. Centrifuge machine, F. Interior of centrifuge machine, G. Platelet-rich fibrin (PRF) in test tube, H. PRF clot.

The periodontal examination showed a normal probing depth for #21 and adjacent teeth. There was no response to cold test with Endo-Frost (Coltene/Whaledent AG, Altstaetten, Switzerland) or electric pulp tester (Parkell Inc., Edgewood, NY, USA) on #21 while there was a positive response to the same with the adjacent teeth. There was no tenderness on percussion. Radiographic examination of #21 showed periapical rarefaction along with a 3 mm open apex which was confirmed using Image J software (National Institute of Mental Health, Bethesda, MD, USA) (Figure 1A). Based on clinical and radiographic examination, we arrived at a diagnosis of pulpal necrosis and asymptomatic apical periodontitis with respect to #21. The different treatment options were explained to the patient and his mother who consented for regenerative endodontic procedures with the use of autologous PRF as a scaffold. The methodology for regenerative endodontic procedures was based on the guidelines of the American Association of Endodontists and the European Society of Endodontology.

Treatment for tooth no #21 was initiated with the administration of 2% lignocaine with 1:200000 adrenaline and rubber dam isolation. After access opening and working length determination (Figure 1B), minimal filing with size #15Hfile (Mani Inc, Utsunomiya, Japan) was done followed by copious irrigation with 20 mL of 0.5% sodium hypochlorite (Prime Dental, Bhiwandi, India) over 5 min followed by 20 mL of 17% ethylenediaminetetraacetic acid (Desmear, Anabond Stedman Pharma Research Ltd., Chennai, India) again over 5 min. After drying the canal, calcium hydroxide (Ultracal XS; Ultradent products, Jordan, UT, USA) was placed as an intracanal medicament. A cotton pellet was placed and the access cavity was sealed with 3-4 mm of Cavit (3M ESPE, St Paul, MN, USA). The patient was recalled after 2 weeks and was asymptomatic with #21, showing no tenderness to palpation and percussion. The tooth was isolated, anesthetized, re-accessed, and calcium hydroxide was removed through the same irrigation protocol as in the previous appointment (Figure 1C).

**Figure 2:**
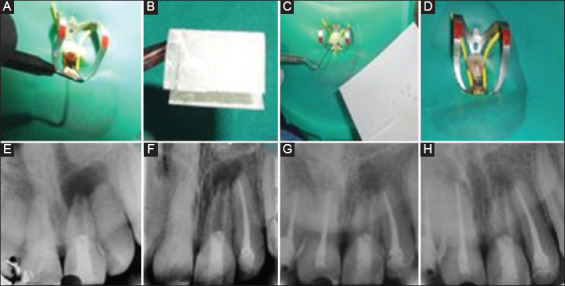
A. Bleeding evoked in canal, B. Collagen matrix, C. Placement of mineral trioxide aggregate, D. Placement of RMGIC and composite, E. 1 month follow-up, F. 6 month follow-up, G. 1 year follow-up, H. 2 year follow-up.

A volume of 10 mL of blood was drawn from the patient’s cubital vein (Figure 1D), collected in a glass test tube, and centrifuged at 400×g (times gravity) for 10 min in a tabletop centrifuge machine (Figure 1E and F) (Model R-8C Plus, Remi Laboratories, Mumbai, India). Choukroun’s PRF was obtained in the test tube (Figure 1G) as the middle layer with acellular plasma at the top and red blood cell at the bottom using sterile tweezers, the fibrin clot was removed (Figure 1H). Intracanal bleeding was intentionally evoked by extending with #80 K-file (Mani Inc, Utsunomiya, Japan) passively about 2 mm past the apical foramen and he blood was allowed to clot in the apical third (Figure 2A). The freshly prepared PRF was then placed into the canal opening, then pushed below the level of the cementoenamel junction using hand pluggers such that it reached to the apical end. Collagen matrix (RTR Membrane, Septodont, Saint-Maur-des-Fossés, France) (Figure 2B) was placed over the PRF to act as a barrier for placement of MTA which was placed 2 mm beneath the cementoenamel junction over the collagen matrix (Figure 2C). Resin modified glass ionomer cement (GC, Tokyo, Japan) was placed as a liner and permanent restoration was done using resin composite (Filtek Z350 XT, 3M ESPE, St Paul, MN, USA ) (Figure 2D).

The patient was followed up at the intervals of 2 weeks, 1 month, 3 months, 6 months, 1 year, and yearly afterward. Intraoral periapical radiographs were taken using a paralleling technique with Rinn XCP receptor holder (Dentsply Rinn, Charlotte, NC, USA) at a constant positive angulation, kilovoltage peak (kVP), milliampere seconds (mAs), and exposure time during each of the follow-up periods. This was done to enable the superimposition of the different radiographs and measure and record changes in the tooth and periapical regions over time.

At 2 weeks and a 1-month follow-up, the patient was asymptomatic with no sensitivity to percussion, palpation, or cold test with Endo-Frost (Coltene/Whaledent AG, Altstaetten, Switzerland). There were no evident radiographic changes also (Figure 2E). The patient did not report for the next few months and reported only for the 6-month evaluation. From the intraoral periapical radiograph, a reduction in the size of the periapical radiolucency could be observed (Figure 2F). However, there was still no response to cold testing.

At 12 months recall visit, the patient complained of swelling in #11, #12, and pain in #22 region. Pulp sensibility test with Endo-Frost (Coltene/Whaledent AG, Altstaetten, Switzerland) and electric pulp tester (Parkell Inc., Edgewood, NY, USA) revealed non-vital response in #11,#12, and #22. Root canal treatment was initiated on that visit for #11, #12, and #22. Calcium hydroxide dressing was given for 2 weeks and the root canal procedure was completed for #11, #12, and #22. Intraoral periapical radiograph revealed partial apical closure of #21 of about 1.5 mm which was confirmed using Image J software (National Institute of Mental Health, Bethesda, MD, USA) (Figure 2G). At the final follow-up which was after 2 years, #21 was functional and asymptomatic. In intraoral periapical radiograph, the apical diameter of #21 was about 1mm and there was a reduction in the size of periapical radiolucency (Figure 2H).

## 3. Discussion

Management of immature permanent teeth with open apices and pulpal necrosis is a significant challenge. Conventionally, the apexification procedure was done in such cases, but the regenerative endodontic procedure has emerged as a valuable alternative later. Teeth with necrotic pulp and an immature apex in which pulp space is not needed for post/core are the indications for the regenerative procedure. A major concern in teeth undergoing regenerative endodontic treatment is achieving optimal disinfection of the root canal system [6]. *In vitro* studies revealed that the use of antibiotics at high concentrations for disinfection has disadvantageous effects on the survival of stem cells and the quality of dentinal walls [7]. Calcium hydroxide was found conducive to stem cell survival, proliferation, and differentiation [8]. Hence, in this case, we placed calcium hydroxide as an intracanal medicament.

The three key elements of tissue regeneration are stem cells, growth factors, and scaffold. Stem cells from apical papilla are thought to be the main cell source for pulp regeneration in immature teeth. Stem cells from apical papilla are referred to as mesenchymal stem cell populations residing at the apical papilla around the root apices of immature permanent teeth [3,9,]. Periodontal ligament stem cells, bone marrow mesenchymal stem cells [10], and some surviving dental pulp stem cells around the root apex may participate in pulp regeneration, but they need to be stimulated to migrate into root canal space. Bleeding induced by passing files beyond the apex was thought to stimulate the migration of adult stem/progenitor cells into the root canal. In our patient, intracanal bleeding was induced by overinstrumenting the canal with hand files to stimulate the migration of stem/progenitor cells. Growth factors promote the differentiation of mesenchymal stem cells into odontoblast like cells. Blood from periapical tissues has been shown to contain mesenchymal stem cell markers and platelet-derived growth factors (PDGF) in high concentrations that facilitate the processes of tissue forming and remodeling [11].

In the case that presented as infected necrotic immature teeth, we used PRF as a scaffolding material for pulpal regeneration and tooth revitalization. PRF was introduced by Choukroun in 2001. The fibrin matrix contains a large number of platelets and cytokines. Fibrin helps in the migration of fibroblasts and endothelial cells and is the source of growth factors that aid in revascularization. PRF has been several advantages over platelet-rich plasma which include ease of preparation and lack of biochemical handling of blood, which makes this preparation strictly autologous [12]. Studies have demonstrated that the PRF has a significant slow, sustained release of many key growth factors like PDGF and transforming growth factor-beta for at least 1 week and up to 28 days, which means that PRF could release growth factors with its biological scaffold [13]. PRF by Choukroun’s technique does not dissolve quickly after application; instead, the fibrin matrix is slowly remodeled similar to the natural blood clot [14].

In our case, the collagen scaffold was used as a matrix and 3 mm of MTA was packed and condensed to obtain a tight coronal seal. MTA by itself provides signaling molecules for the growth of the stem cells [15].

On radiographic examination, we could elucidate that there was continued thickening of the dentinal walls, root lengthening, regression of the periapical lesion, and partial apical closure in 2 years which can be attributed to the use of PRF. The results of our study are in accordance with the study done by Nazzal *et al*. [16] in which apical closure and periodontal healing were observed in traumatized immature teeth with necrotic pulps treated with revitalization endodontic technique. A study revealed that the PRF causes the proliferation of human dental pulp cells and increases the protein expression of osteoprotegerin and alkaline phosphatase activity [17]. Some amounts of human dental pulp cells present in the apical papilla usually remain vital even in case of a large periapical lesion. After the regression of the inflammation and under the influence of Hertwig’s epithelial root sheath, these dental pulp cells differentiate into odontoblasts like cells.

Concerning vitality assessment, there was no positive response to the cold test during the follow-up period and a longer follow-up may be required to observe a positive response and to gain the nerve function after regenerative endodontic procedures. A few studies were able to show a positive response to pulp sensibility tests up to 2-3 years after initiation of treatment, thus indicating revitalization [18,19]. However, the initial outcome assessment criteria of regenerative endodontic therapy in necrotic teeth were largely based on radiographic healing of apical periodontitis and clinical symptoms before a positive response to pulp vitality tests become evident [18]. In the present case, the regenerative endodontic procedures led to the regression of clinical symptoms and also to the reduction in the size of the apical radiolucency. In the future, diagnostic tools for more accurate initial outcome assessment of regenerative endodontic procedures need to be developed.

## 4. Conclusions

Based on the result of our case report, we conclude that the revitalization of a necrotic infected immature tooth is possible under conditions of total canal disinfection and PRF is an ideal biomaterial for regeneration. However, randomized clinical trials are necessary to compare the effect of PRF and induced bleeding on the revitalization of a tooth with necrotic pulp and open apex on a long-term basis.
